# Life‐history traits of a tropical bagrid catfish, *Mystus mysticetus* Roberts, 1992, caught from the Mekong Delta, Vietnam

**DOI:** 10.1002/ece3.10280

**Published:** 2023-07-09

**Authors:** Thu Quynh Phan, Lam Thi Thao Vo, Anh Ngoc Tran, Quang Minh Dinh

**Affiliations:** ^1^ Department of Biology School of Education Can Tho University Can Tho Vietnam

**Keywords:** exploitation rate, length‐frequency, longevity, mortality, *Mystus mysticetus*

## Abstract

Population's biological parameters, including length at first capture, mortalities, exploitation rates, growth coefficient, longevity, and recruitment times, are essential in assessing fishing status, but there is no data on *Mystus mysticetus*. Therefore, the study was conducted to provide these parameters to assess the fishing status of this species at Cai Rang, Can Tho (CRCT) and Long Phu, Soc Trang (LPST). A collection of 741 individual fish was used for analysis and showed that most fish size groups ranged from 9.0 cm to 12.0 cm, and the asymptotic length was 16.8 cm for both CRCT and LPST populations. The fish population von Bertalanffy curve was *L*_t_ = 16.80(1 − e^−0.51(*t* + 0.38)^) at CRCT and *L*_t_ = 16.80(1 − e^−0.48(*t* + 0.40)^) at LPST. The fish growth coefficient at CRCT (2.16) was higher than at LPST (2.13), whereas the reverse case was true for longevity ranging from 5.88 years (at CRCT) to 6.25 years (at LPST). At CRCT, fishing mortality, natural mortality, total mortality, and exploitation rate were 0.69/year, 1.40/year, 2.09/year, and 0.33, respectively; at LPST, these values were 0.75/year, 1.33/year, 2.08/year, and 0.36, respectively. Although the population parameter of this fish species exhibited a spatial variation, both CRCT and LPST fish resources have not been subjected to overexploit because *E* (0.33 at CRCT and 0.36 at LPST) is lower than *E*_0.1_ (0.707 at CRCT and 0.616 at LPST).

## Graphical abstract

The fish population von Bertalanffy curve was Lt = 16.80(1 − e‐0.51(t + 0.38)) at CRCT and Lt = 16.80(1‐ e‐0.48 (t + 0.40)) at LPST; and the fish growth coefficient at CRCT (2.16) was higher than at LPST (2.13), whereas the reverse case was true for lifespan ranging from 5.88 years (at CRCT) to 6.25 years (at LPST). At CRCT, fishing mortality, natural mortality, total mortality, and exploitation rate were 0.69/year, 1.40/year, 2.09/year, and 0.33, respectively; at LPST, these values were 0.75/year, 1.33/year, 2.08/year, and 0.36, respectively. Although the population parameter of this fish species exhibited a spatial variation, both CRCT and LPST fish resources have not been subjected to overexploit because E (0.33 at CRCT and 0.36 at LPST) is lower than E_0.1_ (0.707 at CRCT and 0.616 at LPST).

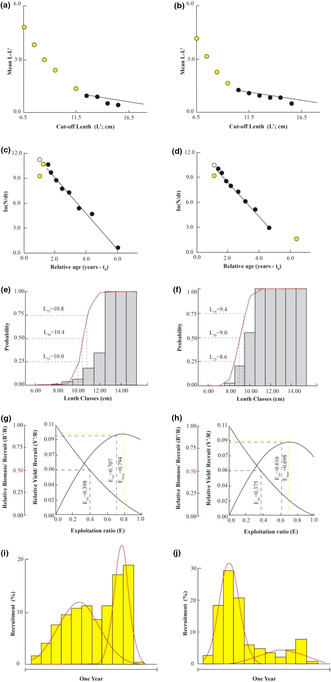

## 1 INTRODUCTION

*Mystus mysticetus*, a member of the Bagridae family, is represented by three dark longitudinal stripes running vertically below the lateral line, a dark black spot at the back of the operculum, a gray‐black margin, an adipose dorsal fin that is short‐tall and quite a distance from the dorsal fin (Phan et al., 2022; Tran et al., 2013; Vo et al., 2021). The *Mystus* is of genera of the family Bagridae, a family of fish native to Africa and Asia (Berg, 1858), with about 21 genera and 89 species (Fricke et al., 2022). In Vietnam, species of the genus *Mystus* are concentrated in the Mekong Delta (VMD) and some other localities in the central and northern regions of Vietnam. According to FAO, in the Mekong River basin, there are 13 species of the genus *Mystus*, typically *M. atrifasciatus* (Fowler, 1937), *M. vittatus* (Bloch, 1794), *M. gulio* (Hamilton, 1822), *M. wolffii* (Scopoli, 1777), *M. rhegma* (Roberts, 1994), *M. planiceps* (Valenciennes, 1840), and *M. albolineatus* (Robert, 1994) (Tran et al., 2013). They live at the bottom of freshwater bodies, migrating into flooded forests during the wet season and returning to the river's lower reaches in November and December of the lunar calendar (Rainboth, 1996; Vo et al., 2023). That migration feature has created a difference in the habitat of *M. mysticetus* and may lead to the ability to change morphology to adapt to different habitats (Nguyen & Duong, 2020; Vo et al., 2021). *Mystus mysticetus* is one of the target‐catching catfish and plays a vital role in the local food supply (Vo et al., 2023).

Aquatic resources are essential to the lives of communities worldwide, and one of the crucial tasks of studying population variability is estimating the population's parameters, such as abundance, growth, recruitment period, mortalities, length at first capture, longevity, and exploitation rate (Tran, 2010). These parameters are used to assess the fishing status of the fish population (Amezcua et al., 2006), but the data on *M. mysticetus* populations along the Hau River, where they are fishing for food provision, is limited. Cai Rang, Can Tho (CRCT) has favorable climatic and hydrological conditions with fresh water all year round, whereas the Long Phu, Soc Trang (LPST) is affected by saline intrusion in the dry season (Nguyen et al., 2021). This phenomenon regulates the variation in population biological parameters of fish, for example, *Glossogobius sparsipapillus* exhibits significant differences in growth performance; longevity; total, natural, and fishing mortalities between CRCT and LPST (Nguyen et al., 2021). Therefore, this study aimed to provide parameters such as first catch length, longevity, growth coefficient, exploitation rate, mortalities, and recruitment time of this species and verify if these parameters in CRCT and LPST exhibit a variation. These parameters are the basis for fish ecological adaptation and fishing assessment to set up a reasonable exploitation strategy for this fish resource in CRCT and LPST.

## 2 MATERIALS AND METHODS

### 2.1 Sampling site and fish collection

**FIGURE 1:**
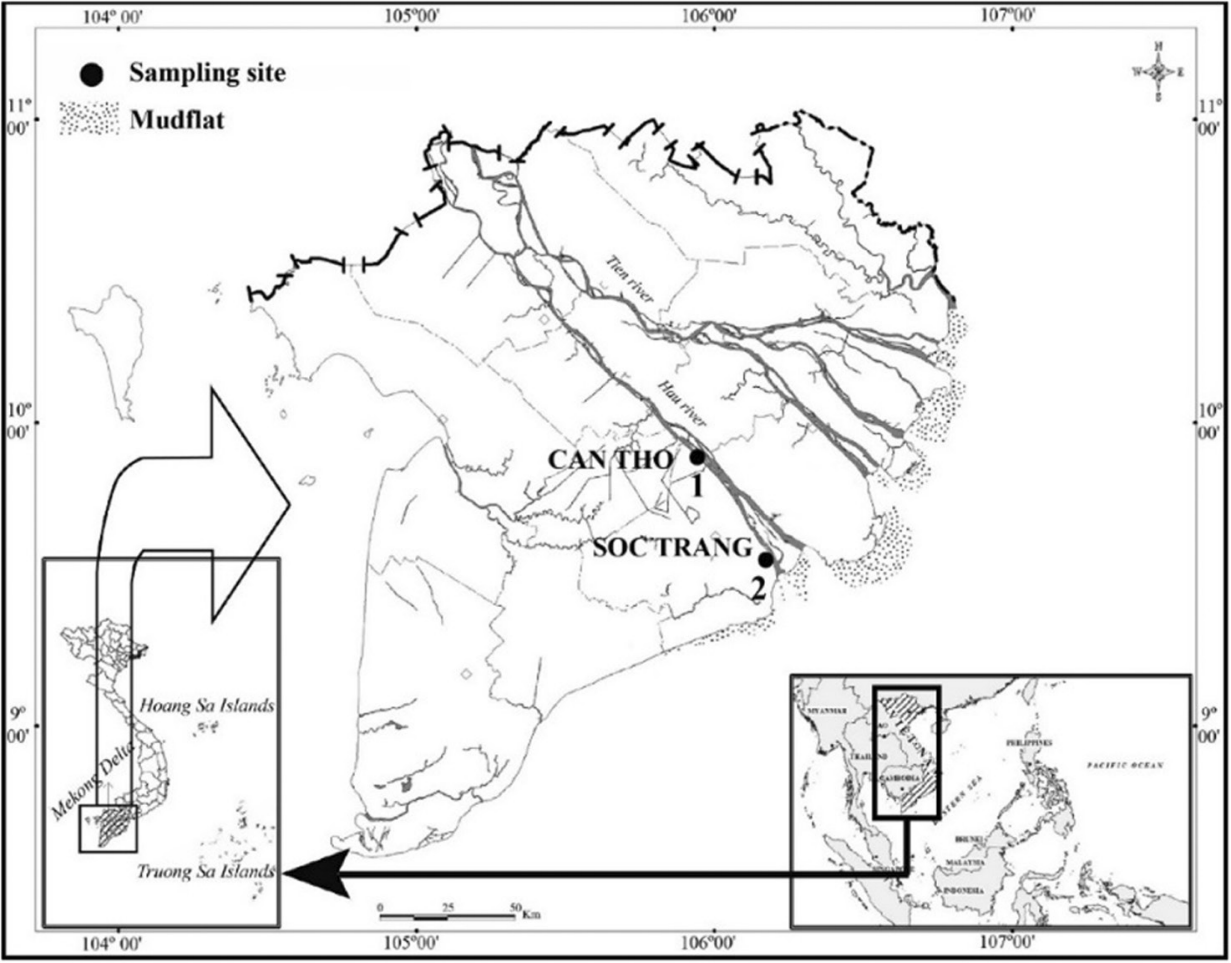
Sampling sites in the Mekong Delta (1: Cai Rang, Can Tho; 2: Long Phu, Soc Trang; *Source*: Dinh (2018)).

The study was carried out at two sites along the Hau River: CRCT, which is freshwater, and LPST, which is brackish water (Figure 1) because *M. mysticetus* displayed a wide distribution from freshwater to salted water. Each month, *M. mysticetus* samples were collected using trawl nets with a size length of 15 m, height of 3 m, and code‐end mesh of 2a = 1.5 cm from January 2022 to December 2022 with ~30 fish samples/month. Each sampling period lasted 2 days, and nets were set up during high tide and retrieved during low tide at each sampling site, where the pH, temperature, and salinity were also measured using the HI98107 tool (pH and temperature) and a refractometer 950.0100 PPT‐ATC (salinity). The fish samples, after collection, were identified based on the morphological characteristics described by Tran et al. (2013) and transported to the Laboratory for further analysis. Fish samples were measured in total length (*L*, cm) to determine the length frequency.

### 2.2 Data analysis

The population biological parameters were determined through length frequency data of males and females at two sites. Male and female data were combined at each location to ensure an adequate sample size. The procedure was performed as described by Amarasinghe and De Silva (1992) to minimize length‐frequency bias due to gear selection's effect. The Powell‐Wetherall approach was applied to estimate the initial *L*_∞_ via the linear regression: *L*_m_ − *L'* = *a + bL* (*L'*: the cut‐off length; *L*_m_: the mean length of all fish; *b*: the slope; *a*: the intercept; *L*_∞_ = *a/b*) (Wetherall, 1986). Next, the ELEFAN I procedure was performed to determine the initial growth parameter (*K*) from the fish's initial *L*_∞_ and length frequency (Gayanilo et al., 2005).

The growth coefficient (Φ') was estimated from the formula suggested by Pauly and Munro (1984): Φ2 = log*K* + 2log*L*_∞_. This coefficient was species‐specific and is used to compare the Φ' of the same species but distributed in different regions or between species within a genus or subfamily or a family with the same distribution based on the research method proposed by Tran et al. (2007).

The longevity (*t*_max_) was determined as *t*_max_ = 3/*K* (Pauly, 1980; Taylor, 1958). The yield curve converted routine was applied to determine the total mortality (*Z*) (Pauly et al., 1995). The natural mortality (*M*) was determined by the formula of Pauly (1980): Log*M* = −0.0066‐0.279 × log *L*_∞_ + 0.6543 × log*K* + 0.463 × log*T*. According to Ricker (1975), fishing mortality (*F*)—the number of individual fish that die directly or indirectly due to fishing activity—was calculated as *F* = *Z–M*; and the exploitation rate (*E*) was determined as *E* = *F/Z*.

The length at which 50% of the fish were caught was called the first catch length (*L*_c_) and was determined by the yield curve transformation equation (Pauly, 1987). The yield/recruitment (Y′/R) and biomass/recruitment (B′/R) models of Beverton and Holt (1957) were used to estimate the maximum exploitation coefficient (*E*_max_), optimal mining factor (*E*_10_), and mining factor at which B′/R was reduced by 50% (*E*_50_). Based on the research method of Pauly and Soriano (1986), the combination of isopleth (*L*_c_/*L*_*∞*_) and exploitation rate (*E*) was used to determine the fishing status of the fish.

The above population biological parameters were obtained by performing FiSAT II software. The temperature, pH, and salinity variation between the dry and wet seasons and sampling sites were quantified using one‐way ANOVA performed by SPSS v.21 at a significance level of 5% (please find the result in the Appendix S1).

## 3 RESULTS

The length frequency data of this fish were determined based on the results of measuring the length of 741 individuals collected at CRCT and LPST. The smallest individual was 7.7 cm, while the longest was 16.8 cm. At LPST, the lengths of the individuals were divided into five groups, in which, the size with the most individuals was 9–10 cm (47 individuals), 10–11 cm (53 individuals), 11–12 cm (78 individuals), 12–13 cm (68 individuals), and 13–14 cm (68 individuals). Similar length frequencies were also found in the LPST population; however, the common length was concentrated in groups of 10–11 cm (102 individuals) and 11–12 cm (98 individuals).

**FIGURE 2:**
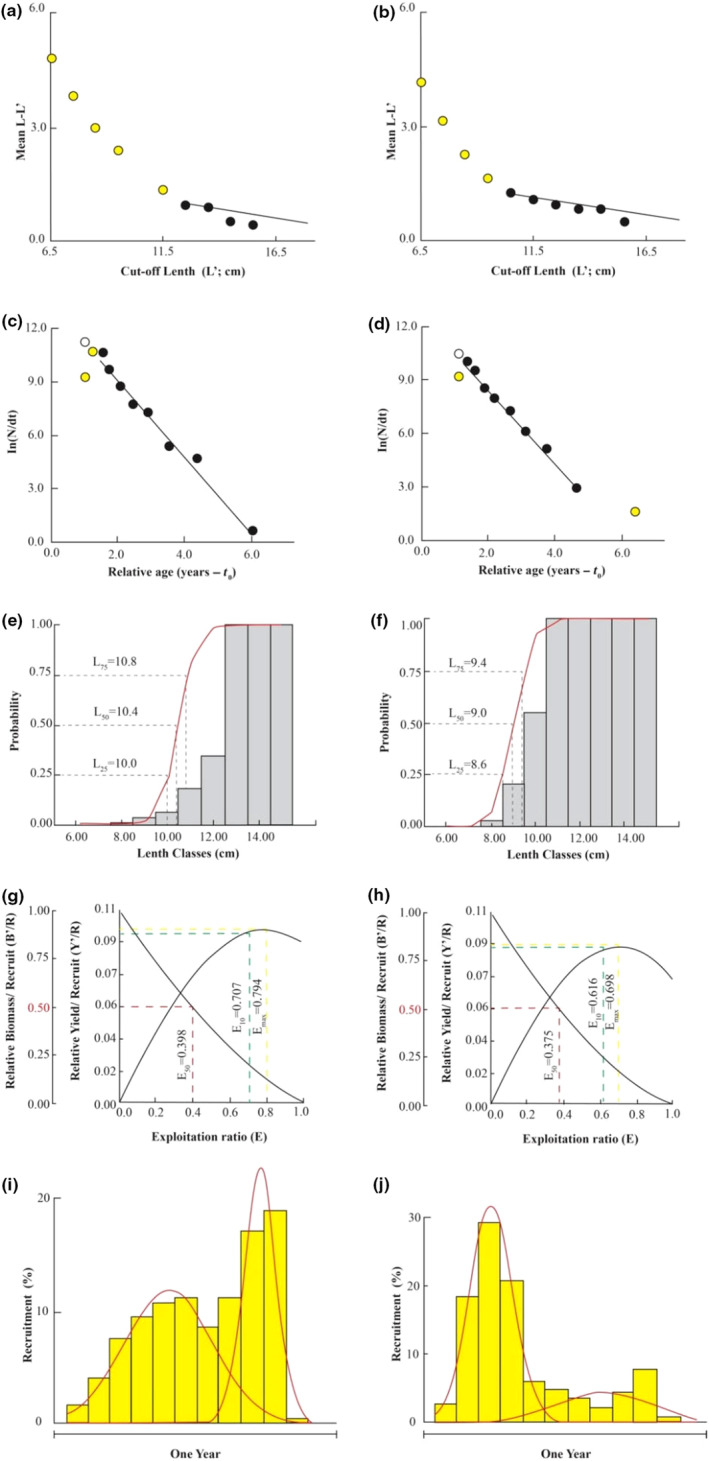
The plots of the Powell‐Wetherall model of *Mystus mysticetus* at CRCT (a) and LPST (b); length converted catching curve of *M. mysticetus* at CRCT (c) and LPST (d); the probability of capture of *M. mysticetus* at CRCT (e) and LPST (f); the relative yield/recruit and biomass/recruit of *M. mysticetus* at CRCT (g) and LPST (h); the recruitment time of *M. mysticetus* at CRCT (i) and LPST (j) (CRCT: Cai Rang, Can Tho; LPST: Long Phu, Soc Trang).

**FIGURE 3:**
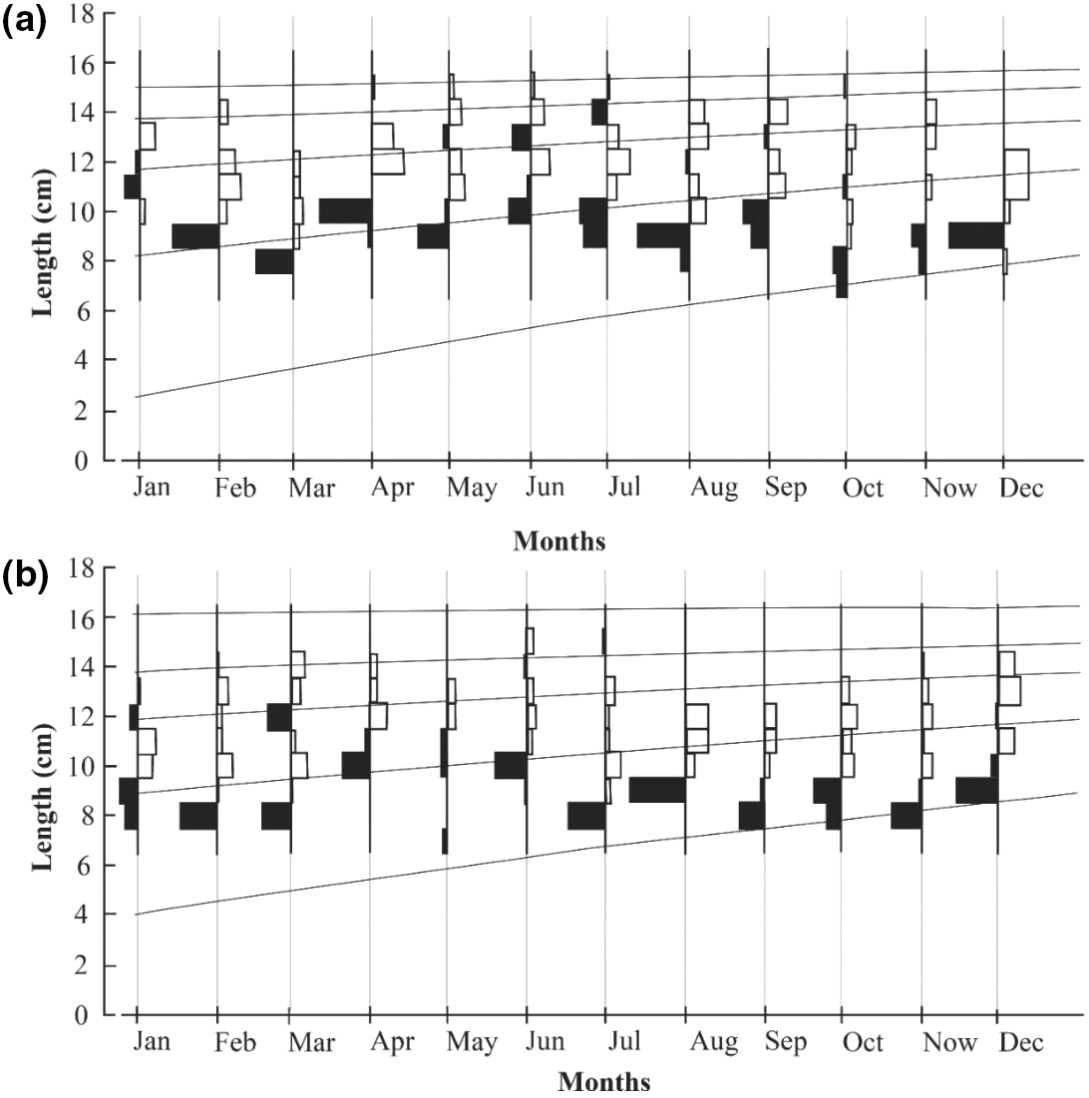
Growth curves of *Mystus mysticetus* estimated using ELEFAN I (a: Cai Rang, Can Tho; b: Long Phu, Soc Trang).

The Powell‐Wetherall plot showed the fishing stages of fish populations at CRCT (Figure 2a) and LPST (Figure 2b) and gave reasonable results of *L*_∞_ (18.37 cm at CRCT and 19.12 cm at LPST) and *Z/K* (4.689 at CRCT and 5.953 at LPST). The growth curves of *M. mysticetus* at CRCT and LPST were shown in Figure 3. Analytical results showed that the population growth parameters of this species at CRCT were *L*_∞_ = 16.80 cm; *K* = 0.51/year, *t*_0_ = −0.38 year, and the von Bertalanffy growth curve equation was *L*_t_ = 16.80(1 − *e* ^*−*0.51(t + 0.38)^). Meanwhile, these parameters of LPST population were *L*_∞_ = 16.80 cm; *K* = 0.48/year, *t*_0_ = −0.40 year, respectively, and the growth curve equation was *L*_t_ = 16.80(1 − *e* ^*−*0.48(t + 0.40)^).

The yield curve converted from fish length frequency analysis showed that the fishing mortality, natural mortality, total mortality, and exploitation rate of the CRCT population were 0.69/year, 1.40/year, 2.09/year, and 0.33, respectively (Figure 2c). These parameters of the LPST population were 2.08/year, 1.33/year, 0.75/year, and 0.36, respectively (Figure 2d). The first catch length (*L*_c_ or *L*_50_) of the fish population at CRCT was 10.4 cm (Figure 2e) which was longer than that at LPST (9.0 cm) (Figure 2f).

The yield/recruitment and biomass/recruitment analysis results indicated that *E*_max_, *E*_10_, and *E*_50_ were 0.794, 0.707, and 0.398 at CRCT (Figure 2g). These values exhibited a lower at LPST, being 0.698, 0.616, and 0.375, respectively (Figure 2h). The *Φ'* showed a greater value in CTCT population (2.158) than in LPST (2.132), whereas the reverse case was found in *t*_max_ ranging from 5.88 years at CRCT to 6.25 years at LPST. The fish isopleth (*L*_c_/*L*_∞_) was 0.620 at CRCT and 0.535 at LPST. This species displayed two recruitment times: June and October (at CRCT) and March and October (at LPST), but the prominent peak of recruitment was different in October at CRCT (Figure 2i) and in March at LPST (Figure 2j).

## 4 DISCUSSION

The *Φ'* of the CRCT population (2.158) was higher than that of the LPST population (2.132) could be due to differences in maximum length between these species. This variation could be related to the variation of temperature, pH, and salinity between these two sites, which was found in Pauly and Munro (1984), who indicated that environmental factors could affect the fish growth coefficient (*Φ'*). One of its congeners, *M. gulio* in Bangladesh, displayed a higher value (2.599) than that of *M. mysticetus* (Mustafa et al., 2019), which might be because *M. gulio* had a higher *L*_∞_ (23.0 cm) than *M. mysticetus*. Compared with some previous studies of fish of the family Bagridae, *M. mysticetus* in the present study grew more slowly than them, for example, *Hemibagrus nemurus* (2.808) in Indonesia (Rosadi et al., 2020), *Chrysichthys nigrodigitatus* (2.968) in Côte d'Ivoire (Bédia et al., 2017), *C. nigrodigitatus* (2.867) in Nigeria (Ikongbeh et al., 2015), *C. nigrodigitatus* (3.12), *Chrysichthys auratus* (2.76) in Ghana (Ofori‐Danson et al., 2002), *C. auratus* (2.18) in Egypt (Ragheb, 2014).

Although located in the Mekong Delta, in each ecological region, fish had different growth rates (Dinh, Nguyen, Nguyen, et al., 2022). Indeed, the growth parameter (*K*) of *M. mysticetus* at CRCT (0.51/year) was higher than at LPST (0.48/year), whereas the opposite case was found for maximum life with 5.88 years at CRCT and 6.25 years at LPST. *M. mysticetus* displayed a higher *t*_max_ than *M. mysticetus* like *M. gulio* (4.0 years) (Mustafa et al., 2019) and other Bagridae species, such as *H. nemurus* (3.75 years) in Indonesia (Rosadi et al., 2020), *C. nigrodigitatus* (5.66 years) in Nigeria (Ikongbeh et al., 2015), *C. nigrodigitatus* (4.62 years), and *C. auratus* (5.0 years) in Ghana (Ofori‐Danson et al., 2002). However, *M. mysticetus* exhibited lower *t*_max_ than that of *C. nigrodigitatus* (9.09 years) in Côte d'Ivoire (Bédia et al., 2017) and *C. auratus* (14.02 years) in Egypt (Ragheb, 2014). The variation of *K* among these species could be related to the different place fish collection.

The *M* of *M. mysticetus* was higher than that of *C. nigrodigitatus* (0.73/year) in Côte d'Ivoire (Bédia et al., 2017), *C. nigrodigitatus* (1.051/year) in Nigeria (Ikongbeh et al., 2015), *C. nigrodigitatus* (1.24/year) and *C. auratus* (1.30/year) in Ghana (Ofori‐Danson et al., 2002), *C. auratus* (0.6/year) in Egypt (Ragheb, 2014). It seems that *M. mysticetus* could be more sensitive to environmental conditions than other fishes in the Bagridae family. Meanwhile, the *F* of *M. mysticetus* was lower than that of *M. gulio* (1.42/year) in Bangladesh, *H. nemurus* (0.83/year) in Indonesia (Rosadi et al., 2020), *C. nigrodigitatus* (1.73/year) in Côte d'Ivoire (Bédia et al., 2017), *C. nigrodigitatus* (2.53/year) and *C. auratus* (1.33/year) in Ghana (Ofori‐Danson et al., 2002), *C. auratus* (0.84/year) in Egypt (Ragheb, 2014). The cause of this difference could have been the difference in economic value and the diversity of fishing gear.

At CRCT and LPST, the *E* of *M. mysticetus* was lower than *E*_10_, suggesting that the fish stocks in these regions were not subjected to overexploited. The fish age at LPST tended to be caught earlier than at CRCT, as *L*_c_/*L*_∞_ at LPST (0.535) was lower than at CRCT (0.620). Likewise, studies on several species of Bagridae shared similar results, for example, *M. gulio* in Bangladesh (Mustafa et al., 2019) and *C. auratus* in Egypt (Ragheb, 2014). On the other hand, some populations of Bagridae are overexploited, such as *C. nigrodigitatus* in Côte d'Ivoire (Bédia et al., 2017) and *C. nigrodigitatus* in Ghana (Ofori‐Danson et al., 2002).

The variations in temperature, pH, and salinity at CRCT and LPST seem to cause significant differences in population biological parameters in this fish. In which, the salinity could be the most substantial influencing factor for parameters such as longer longevity at LPST and higher growth coefficient at CRCT. However, some morphological parameters showed stability with the environment of this fish as the maximum length remains unchanged, and the exploitation rate was similar. With harsher conditions at LPST, the natural mortality in this area (0.75/year) was significantly higher than that at CRCT (0.69/year). Sudden changes in salinity conditions could lead to fish at increased mortality risk or poorer vitality. The difference in these two regions affected not only the population of this fish but also many other fish species in the same distribution area. In the Gobiidae family, *G. sparsipapillus* showed this difference (Nguyen et al., 2021). Specifically, in this fish, there was a difference in the sex ratio at CRCT (females and males were equal in number) and LPST (males predominate in number). The *t*_max_ at CRCT displayed a higher value than that of LPST, but the opposite result was found in *Φ'*. Like *M. mysticetus*, *G. sparsipapillus* had an equivalent maximum length between these two regions. Some other fish species also displayed a variation in population parameters regarding ecological regions, for example, *Periophthalmus chrysospilos* exhibited a better adaptation to areas with low salinity (Ben Tre and Tra Vinh) than to areas with higher salinity (Soc Trang and Bac Lieu) (Dinh, Nguyen, Nguyen, et al., 2022). Meanwhile, *Acentrogobius viridipunctatus* displayed a higher growth coefficient in the high salinity areas (Bac Lieu and Ca Mau) (Dinh, Nguyen, Truong, & Nguyen, 2022). That showed environmental conditions in different environments could affect some fish population parameters.

## 5 CONCLUSION

*Mystus mysticetus* has been sampled at a typical length of 10–12 cm, and the von Bertalanffy curve was *L*_t_ = 16.80(1 *− e* ^*−*0.51(t + 0.38)^) at CRCT and *L*_t_ = 16.80(1 *− e* ^*−*0.48(t + 0.40)^) at LPST. The longevity was 5.88 years at CRCT and 6.25 years at LPST, and the growth coefficient was 2.16 at CRCT and 2.13 at LPST. The natural and fishing mortalities were 1.40/year and 0.69/year at CRCT, and 1.33/year and 0.75/year at LPST, respectively. *E*_max_, *E*_10_, and *E*_50_ values were 0.794, 0.707, and 0.398 at CRCT and 0.698, 0.616, and 0.375 at LPST, respectively. These population biology parameters have helped better understand this species' fishing status in the Mekong Delta. Fish populations in the two areas are still exploited reasonably, although their population showed a spatial change. However, limiting fishing during population replenishment, increasing the catch size, and increasing the mesh size of fishing gear are necessary for rational exploitation and sustainable development of this fish resource. *M. mysticetus* has potential in aquaculture production and fishing as population replenishment occurs twice yearly in both ecoregions.

## AUTHOR CONTRIBUTIONS

**Thu Quynh Phan:** Conceptualization (equal); funding acquisition (equal); investigation (equal); methodology (equal); resources (equal); validation (equal); writing – original draft (equal); writing – review and editing (equal). **Lam Thi Thao Vo:** Conceptualization (equal); funding acquisition (equal); investigation (equal); methodology (equal); resources (equal); validation (equal); writing – original draft (equal); writing – review and editing (equal). **Anh Ngoc Tran:** Conceptualization (equal); funding acquisition (equal); investigation (equal); methodology (equal); resources (equal); validation (equal); writing – original draft (equal); writing – review and editing (equal). **Quang Minh Dinh:** Conceptualization (equal); investigation (equal); methodology (equal); supervision (equal); validation (equal); writing – original draft (equal); writing – review and editing (equal).

## FUNDING INFORMATION

This work is supported by Can Tho University under grant number TSV2022‐137.

## CONFLICT OF INTEREST STATEMENT

The authors declare that they have no competing interests.

## Supporting information


Appendix S1

## Data Availability

Data will be provided when you require it.
