# Supplementary material for: Life‐history traits of a tropical bagrid catfish, Mystus mysticetus Roberts, 1992, caught from the Mekong Delta, Vietnam
Source: Ecol Evol. 2023 Jul 9;13(7):e10280. doi: 10.1002/ece3.10280 (PMC10329934; doi:10.1002/ece3.10280)
Supplement: Supplementary file 1 — Appendix S1 [file ECE3-13-e10280-s001.docx]

**Variation of temperature, pH and salinity between CRCT (Cai Rang – Can Tho) and LPST (Long Phu – Soc Trang)**

The temperature survey results showed that the temperature in the dry season was significantly higher than in the rainy season (F=35.03, p<0.01). The average temperature values in the dry and rainy seasons were 30.54±0.112 ^o^C SE and 29.67±0.10^o^C SE, respectively. Similar to temperature, the pH in the dry season (7.74±0.03 SE) was also higher than that in the rainy season (7.66±0.02 SE) (F=4.92, p=0.04). Because saline intrusion occurs at LPST during the dry season months, the mean value of salinity in these two study sites in the dry season (6.30±0.64‰ SE) was significantly higher than that in the rainy season (1.21±0.54‰ SE) (F=36.39, p<0.01).

The results also showed that the temperature at LPST gave a significantly higher value than CRCT (F=11.60, p=0.003). The temperature values at these two sites were 30.36±0.10^o^C SE and 29.86±0.10^o^C SE, respectively. In contrast, the pH at LPST (7.65±0.02 SE) was significantly lower than at CRCT (7.74±0.03 SE) (F=5.99, p=0.02). In all months, the salinity at CRCT was recorded as zero, while the mean salinity value in the studied months at LPST was 7.51±0.60‰ SE (F=79.45, p<0.01).

Season and site interactions did not affect temperature (F=0.46, p=0.50) and pH (F=1.90, p=0.18). In contrast, season and site interactions affected salinity (F=36,39, p<0.01).
